# Prospective association of the SHARE-operationalized frailty phenotype with adverse health outcomes: evidence from 60+ community-dwelling Europeans living in 11 countries

**DOI:** 10.1186/1471-2318-13-3

**Published:** 2013-01-03

**Authors:** Nejma S Macklai, Jacques Spagnoli, Julien Junod, Brigitte Santos-Eggimann

**Affiliations:** 1 Institute of Social and Preventive Medicine (IUMPS), Lausanne University Hospital, 10 route de la Corniche, 1010, Lausanne, Switzerland

**Keywords:** Frailty phenotype, Validation, Adverse outcomes, Population survey, SHARE, BADL disability, IADL disability, Morbidity

## Abstract

**Background:**

Among the many definitions of frailty, the frailty phenotype defined by Fried et al. is one of few constructs that has been repeatedly validated: first in the *Cardiovascular Health Study* (CHS) and subsequently in other large cohorts in the North America. In Europe, the *Survey of Health, Aging and Retirement in Europe* (SHARE) is a gold mine of individual, economic and health information that can provide insight into better understanding of frailty across diverse population settings. A recent adaptation of the original five CHS-frailty criteria was proposed to make use of SHARE data and measure frailty in the European population. To test the validity of the SHARE operationalized frailty phenotype, this study aims to evaluate its prospective association with adverse health outcomes.

**Methods:**

Data are from 11,015 community-dwelling men and women aged 60+ participating in wave 1 and 2 of the *Survey of Health, Aging and Retirement in Europe*, a population-based survey. Multivariate logistic regression analyses were used to assess the 2-year follow up effect of SHARE-operationalized frailty phenotype on the incidence of disability (disability-free at baseline) and on worsening disability and morbidity, adjusting for age, sex, income and baseline morbidity and disability.

**Results:**

At 2-year follow up, frail individuals were at increased risk for: developing mobility (OR 3.07, 95% CI, 1.02-9.36), IADL (OR 5.52, 95% CI, 3.76-8.10) and BADL (OR 5.13, 95% CI, 3.53-7.44) disability; worsening mobility (OR 2.94, 95% CI, 2.19- 3.93) IADL (OR 4.43, 95% CI, 3.19-6.15) and BADL disability (OR 4.53, 95% CI, 3.14-6.54); and worsening morbidity (OR 1.77, 95% CI, 1.35-2.32). These associations were significant even among the prefrail, but with a lower magnitude of effect.

**Conclusions:**

The SHARE-operationalized frailty phenotype is significantly associated with all tested health outcomes independent of baseline morbidity and disability in community-dwelling men and women aged 60 and older living in Europe. The robustness of results validate the use of this phenotype in the SHARE survey for future research on frailty in Europe.

## Background

Associated with old age, but a unique entity, frailty is conceptualized as a state in which reserve function across multiple physiologic domains decline, compromising the individuals’ capacity to withstand stress, thereby predisposing them to poor health, functional decline, institutionalization and death [1-6]. Due to the complex multi-system and heterogeneous nature of the concept, frailty definitions abound [7]. There is no clear consensus on how to operationalize it [8-10] and the lack of a gold standard impedes comparisons across time, countries, and populations in a reliable manner.

Among the many biological, physiological, social and psychosocial models on the possible causes and pathways of frailty [1,11-18], the one that has attracted considerable attention is the “phenotype of frailty” [1]. This concept embodies a set of signs and symptoms that is well aligned with geriatric syndromes, underscoring a biological origin, and reflecting a syndromic character in which an aggregate of multiple-system impairments that are inter-related cause a loss of function [19-21]. As one of most widely-referenced constructs, the frailty phenotype is defined by the presence of three or more of the five specific measurable attributes: weight loss, muscle weakness, poor endurance, slow motor performance and reduced physical activity [1].

Using data from the *Cardiovascular Health Study* (CHS), Fried et al. operationalized the phenotype and confirmed its validity by showing association with 3 and 7-year incidence of mobility and ADL disability, independent of comorbidity among community-dwelling men and women aged 65 years and older [1]. Subsequently, numerous validation studies, including two large cohort studies carried out by Woods et al. [22] and Bandeen-Roche et al. [23], used the same construct with some modifications in the metric used to define each frailty criteria. In spite of these differences, the frailty criteria persisted in showing strong independent associations to poor health outcomes. By testing the criteria and their modifications in different populations these studies substantiate, to a greater extent, the generalizability of the frailty phenotype in the North America populations.

In Europe, the large multi-country and ongoing population-based panel survey of health, social, and economic well-being of community dwellers aged 50 and older, entitled Survey of Health, Aging and Retirement in Europe (SHARE) offers an important opportunity to investigate the disparities of health and their determinants. With as many as 20 countries of the European Union participating, SHARE’s rich and detailed multidisciplinary data source is a promising medium to explore the relationship between frailty and its diverse environment in Europe.

With this in view, using data from SHARE, Santos-Eggimann and colleagues [24] operationalized the Fried frailty criteria by selecting the most suitable metric available and applied it to the first wave of SHARE in order to quantify the burden of frailty across populations in Europe. Since there was considerable divergence from the metric used in the CHS study to define the frailty phenotype, it was necessary to test the SHARE-adapted metric’s prospective association with adverse outcomes in the SHARE population. Advancing on the work of Santos-Eggimann and colleagues, Romero-Ortuno and colleagues used the SHARE - operationalized phenotype and successfully demonstrated its validity in predicting mortality [25].

Testing and validating the operationalization of sound conceptual models is a necessary first step in assessing its reproducibility and reliability in diverse populations for cross country comparisons to be made possible overtime. Hence, to further the confidence in the validity of the SHARE metric, this paper assesses, among the survivors, the prospective association of the frailty criteria, as operationalized in SHARE, on incident mobility, BADL and IADL disability, and on worsening mobility, disability and morbidity over a 2-year follow-up in community dwelling women and men aged 60 and older taking part in the SHARE survey.

## Methods

### Data source and sample

Data are derived from (SHARE, *release 2.4.0)* 11 countries of the European Union (Austria, Belgium, Denmark, France, Germany, Greece, Italy, The Netherlands, Spain, Sweden, and Switzerland) that participated in the 2004 and 2006 wave of SHARE. Representative samples of non-institutionalized population aged 50 years and older [26] were drawn from national or regional population based registries using simple or multistage probably sampling techniques, depending upon the institutional framework of each country. Data for wave1 were collected in 2004 and followed up in 2006 for wave2 using computer-assisted personal interview technique (CAPI) by trained interviewers. Mean response rates were 62%. Detailed description of the SHARE Methodology is documented on the SHARE website [27].

The study eligibility criteria was set up to include all individuals who had participated in both the baseline interview and consented to follow up; born prior to 1945; and reported to be living at home at the time of the interview in 2004. Of the 18, 105 non-institutionalized individuals aged 60+ who participated in 2004, 539 were reported deceased, 5,966 were lost to follow up, and 585 refused further participation in 2006, resulting in a study sample of 11,015 individuals.

#### Frailty definition

Frailty was operationalized using variables in SHARE [24] that approximated those used in the original CHS defining the frailty phenotype construct. *Muscle weakness* was measured using a dynamometer, using the highest of 4-measurement readings (2 from each hand) of handgrip strength after adjusting for gender and body mass index cutoffs as specified by L. Fried [1]. *Exhaustion* criterion was met if the participant answered “yes” to the self-reported question: “In the last month have you had too little energy to do things you wanted to do?”. *Unintentional weight loss* was operationalized using 2 questions in SHARE: 1) “What has your appetite been like?” and/or 2) “So have you been eating more or less?”. Participants scored positive for the criterion if they answered either “Diminution in desire for food” in response to the first question, or “Less” in response to the second question. *Slowness* was operationalized using two questions: “Because of health problems, do you have difficulty walking 100 m, or climbing one flight of stairs without resting?”. Criterion was met if participants answered positive to either of the two questions. *Low physical activity* was operationalized using the question “How often do you engage in activities that require a low or moderate level of energy such as gardening, cleaning the car or going for a walk?”. Participants scored positive for the criterion if responded “One to three times a month, hardly ever, or never”. Subjects were considered frail if they met three or more of these criteria; prefrail if they fulfilled one or two criteria; and not frail if they met none.

### Outcome measures

Seven outcomes were examined to assess the robustness of the SHARE-operationalized frailty criteria in predicting adverse health in the SHARE sample. A 2-year incidence and a 2-year propensity for “worsening” were computed for each of 3 dimensions of functional disability (mobility, IADL and BADL) as well as a 2-year worsening of morbidity.

#### Functional disability

*Mobility disability* was defined by self-report of difficulty in any one of the 8 upper and lower extremity mobility function tasks on the Rosow - Breslau scale [28]: sitting for 2 hrs; getting up from a chair; climbing several flights of stairs without resting; climbing 1 flight of stairs without resting; stooping or kneeling; reaching or extending arms above shoulder; pulling/pushing large objects, and lifting objects of 5 kg. *IADL disability* was defined by self-report of difficulty in any one of the 7 IADL tasks on the Lawton-Brody scale [29]: using a map; preparing a hot meal; shopping for groceries; using a telephone; taking medications; doing work around the house; and managing money and paying bills. *BADL disability* was defined by self-report of difficulty in any one of the 5 BADL tasks on the Katz ADL scale [30]: dressing; bathing; getting in and out of bed; toileting; and eating and/or cutting up food. In each of these three dimensions, participants were asked: “Please tell me whether you have any difficulty doing each of the everyday activities on the card?”. Difficulties that were anticipated to last less than 3 months were excluded.

*Incident mobility, IADL and BADL disability at 2-year follow* was defined as the development of any “new” reported difficulty observed over the 2 year period, in either of the 3 respective functional disabilities, and computed among only those subjects free of the specific type of disability at baseline. *Worsening of mobility, IADL and BADL disability at 2-year follow* was defined as any increase, with respect to baseline, in one or more number of reported difficulties in mobility, IADL, and/or BADL among subjects not already disabled at baseline in all the tasks of the respective functioning domains. It was calculated by taking the difference between the numbers of difficulties reported at follow-up (2006) and those reported at baseline. It was coded “0” if no change or improvement, and “1” if an increase was observed in the number of difficulties within each specific domain of functional disability.

#### Morbidity

*Morbidity* was defined according to the chronic disease list formulated by Fried and colleagues in the Cardiovascular Health Study [1]. The following 7 self-reported chronic diseases were selected and evaluated: 1) myocardial infarction, congestive heart failure, angina, peripheral vascular disease; 2) stroke; 3) HBP or hypertension; 4) diabetes mellitus; 5) chronic obstructive pulmonary disease; 6) rheumatoid arthritis, and 7) cancer. As participants could answer to more than one concurrent condition, a total count was calculated by summing up the frequency reported by each individual at baseline. *Worsening morbidity at 2-year follow up* was defined as an increase with respect to baseline, in one or more number of chronic conditions observed among individuals who did not already accumulate all 7 diagnoses. It was coded “0” if no change or improvements were observed, and “1” if an increase (difference between follow up and baseline count of > = 1) in the number of chronic conditions were observed.

### Control variables

Sex, age (in years), and income (defined by the purchasing power of parity (PPP) household income adjusted for family size transformed to a logarithmic scale) were considered as potential confounders. Although education was considered, it was not retained due to its high correlation with income. Baseline morbidity was also considered as a control variable, and thus was categorized into 3 groups: no chronic condition; 1 chronic condition, and 2+ chronic conditions (multi-morbidity).

### Statistical analysis

Using STATA statistical software program version 11, all statistical analyses were weighted to account for the individual country’s complex sampling schemes. Four primary sampling units were removed due to the inability to compute variance. Weighted proportions were used to describe baseline characteristics of the sample population and chi-square tests were performed to assess differences between the sexes. Further, bivariate analyses were carried out to assess the relationship between frailty and the 7 outcomes using chi-square tests of independence. Finally, multivariate logistic regression analyses were used to assess the effect of the SHARE-operationalized frailty phenotype on the incidence and worsening of functional capacity as well as on worsening of morbidity at 2-year follow up.

Two multivariate models were computed for each of the 3 incidence outcomes (incident mobility, IADL and BADL disability) to test their independent association with frailty over a 2-year follow up. The first model adjusted for demographic variables of age and sex; the second model further adjusted for income and baseline morbidity. Two multivariate models were also computed for each of the 3 “*worsening”* outcomes (worsening mobility, IADL and BADL disability). The first model adjusted for age, sex and disability-specific domain at baseline; the second model further adjusted for income and baseline morbidity. Finally, 2 additional multivariate models were computed to assess the effect of frailty on *worsening morbidity.* The first model adjusted for age, sex and baseline morbidity; the second fully adjusted model also included income status.

## Results

Table 1 shows the baseline burden of frailty, functional disability and morbidity by sex in 11,015 community dwelling men and women aged 60 years and older living in selected European countries. Mean age was 70 years for men and 71 years for women. The SHARE-operationalized frailty phenotype was assessed in 10,237 participants and classified 46.0% as non-frail, 41.1% as pre-frail, and 12.9% as frail. The sex–specific prevalence showed women to be almost twice as likely to be frail (16.4% vs. 8.6%), but only moderately more likely to be pre-frail (43.4% vs. 38.1%) than men. Women also self-reported to be more functionally dependent and morbid than men – as measured by mobility (67.8% vs. 47.5%), IADL (24.8% vs. 12.9%), BADL (15.0% vs. 10.2%) and multi-morbidity (32.6% vs. 28.1%).

**Table 1:** **Wave 1 population baseline characteristics (weighted proportions**)

**Variable**	**Wave 1**	**ALL**	**Male**	**Female**	**Chi**^**2**^
	**Outcome**	**%**	**%**	**%**	**P Value**
		**(weighted prop)**	**(weighted prop)**	**(weighted prop)**	
Frailty	Not frail	46.0	53.2	40.2	0.000
	Pre-frail	41.1	38.1	43.4	
	Frail	12.9	8.6	16.4	
Age	60-64	26.4	28.8	24.5	0.000
	65-69	23.9	26.2	22.2	
	70-74	19.5	19.9	19.2	
	75-79	15.0	13.8	15.8	
	80-84	10.8	8.2	12.8	
	85+	4.1	3.1	5.6	
Functional status	Mobility (1+)	59.0	47.5	67.8	0.000
	BADL (1+)	12.9	10.2	15.0	0.000
	IADL (1+)	19.6	12.9	24.8	0.000
Morbidity	Morbidity (1)	37.0	37.0	37.0	0.000
	Comorbidity (2+)	30.6	28.1	32.6	

Table 2a shows the overall 2-year incidence of mobility (34.5%), IADL (14.8%) and BADL (8.8%) disability among the SHARE sample. The unadjusted risk of functional disability was greatest among frail, and to a lesser extent pre-frail compared to non-frail individuals. Frail individuals had more than 7-fold risk of developing BADLs disability; more than a 5-fold risk of developing IADL disability, and only a 2.5 fold risk of developing mobility disability compared to non-frail individuals.

**Table 2:** Bivariate relationship between frailty in 2004 and outcomes in 2006

**Wave 2 (2006)**	**Overall**	**Non-frail**	**Pre-frail**	**Frail**	**Chi**^**2**^
a) INCIDENCE^1^	%	%	%	%	p
Mobility	34.5	29.3	43.1	70.5	0.000
IADL	14.8	7.9	18.5	45.3	0.000
BADL	8.8	4.4	9.6	31.6	0.000
b) WORSENING^2^					
Mobility	35.6	28.9	39.0	49.7	0.000
IADL	16.4	7.9	19.0	38.9	0.000
BADL	10.5	19.0	10.9	30.9	0.000
Morbidity	26.3	4.3	26.8	31.8	0.003

^1^Limited to individuals with no prevalent disability at baseline.

^2^Limited to individuals not at the maximum level of disability or morbidity at baseline.

Table 2b shows that among individuals who were not already disabled in all tasks at baseline, the 2-year unadjusted propensity for worsening was consistently higher among frail than pre-frail individuals for mobility (49.7% and 39.0%), IADL (38.9% and 19.0%) and BADL (30.9% and 10.9%) disability, respectively. In contrast, the likelihood of worsening morbidity among individuals who did not report all 7 diagnoses at baseline only slightly declined across the frailty gradient: 31.8% for frail, 26.8% for pre-frail, and 24.3% for non-frail individuals.

Table 3a shows frailty to be independently associated with an increased incident mobility (OR 3.07), IADL (OR 5.52) and BADL (OR 5.13) disability. The pre-frail category was also statistically significant, but with a smaller magnitude of effect. Table 3b shows that over a 2-year follow-up period, frailty was independently associated with increased odds of worsening disability (mobility, IADL and BADL) and of worsening morbidity, after fully adjusting for potential confounders. Frail individuals were around twice as likely as pre-frail individuals to worsen on all three functional disabilities. In contrast, the strength of association between frailty and worsening morbidity was weaker for both frail (OR 1.77) and pre-frail (OR 1.30) individuals, but nonetheless significant.

**Table 3:** Multivariate relationship between frailty in 2004 and outcomes in 2006

			**Pre frail**		**Frail**	
**a) INCIDENCE**^**1**^	**Control variables**	**n**	**OR**	**CI**	**OR**	**CI**
Mobilty	a	4813	1.59	1.31-1.92	4.10	1.42-11.84
	b	4795	1.47	1.21-1.80	3.07	1.02-9.26
IADL	a	8531	2.14	1.73-2.64	6.47	4.49-9.34
	b	8495	1.98	1.60-2.46	5.52	3.76-8.10
BADL	a	9217	1.85	1.41-2.42	6.47	4.56-9.19
	b	9176	1.65	1.25-2.17	5.13	3.53-7.44
**b) WORSENING**^**2**^						
Mobility	c	10138	1.63	1.41-1.87	3.18	2.40-4.22
	d	10094	1.55	1.35-1.78	2.94	2.19-3.93
IADL	e	10177	2.27	1.86-2.77	5.11	3.75-6.99
	f	10133	2.11	1.72-2.60	4.43	3.19-6.15
BADL	g	10187	2.14	1.66-2.77	5.59	3.94-7.96
	h	10143	1.91	1.47-2.49	4.53	3.14-6.54
Chronic Conditions	i	10197	1.33	1.14-1.55	1.88	1.45-2.45
	j	10156	1.3	1.12-1.52	1.77	1.35-2.32

*p values <0.05 significant.

^a^Age and sex.

^b^Age, sex, income and morbility at baseline.

^c^Age, sex, baseline mobility.

^d^Age, sex, baseline mobility, income and baseline morbidity.

^e^Age, sex, baseline IADL.

^f^Age, sex, baseline IADL, income and baseline morbidity.

^g^Age, sex, baseline IADL.

^h^Age, sex, baseline IADL, income and baseline morbidity.

^i^Age, sex, baseline morbidity.

^j^Age, sex, baseline morbidity and income.

^1^Limited to individuals with no prevalent disability at baseline.

^2^Limited to individuals not at the maximum level of disability or morbidity at baseline.

## Discussion

Using pooled data from across 11 European countries, the SHARE-operationalized frailty phenotype showed strong and significant prospective association with functional decline and morbidity in community-dwelling men and women aged 60 and older living in Europe. The findings indicate that even after adjusting for age, sex, income and baseline disability or morbidity, frailty persisted to be independently associated with developing mobility, IADL and BADL disability over a 2-year follow up. The SHARE-operationalized frailty phenotype was also significantly associated with worsening mobility, IADL respectively BADL disability, and chronic diseases independent of age, sex, income, and baseline disability and morbidity over a 2-year follow up. Moreover, the risk of functional decline and morbidity was present even among the pre-frail, but with a slightly lower magnitude of effect. This is consistent with the empirical evidence that the risk of disability and/or morbidity for those presenting with 3 or more frailty components is much higher than when 1 or 2 components are presented, and still greater than when no criteria is present. These results suggest that the metric used in SHARE to operationalize the frailty phenotype construct, as described in the original CHS by Fried and colleagues, is robust in identifying a subgroup of vulnerable older populations at risk for developing adverse outcomes.

The results presented here are in line with previous studies validating the frailty phenotype for a range of adverse outcomes [22,23,31-36]. Data from the Cardiovascular Health Study (CHS) showed frailty and prefrailty to be independently associated with worsening mobility and ADL disability [1]. Similarly data on women aged 65–79 from the WHIOS indicated baseline frailty to independently predict ADL disability for frail and pre-frail individuals after adjustments for socio-demographic, disability and co-morbid conditions [22]. Data from WHASI & II found frailty to be significantly associated with a 3-year incidence of IADL and ADL disability among frail and pre-frail individuals after adjustments [23]. The findings of this study also concur with European studies that have explored frailty in its broader sense in relation to disability, morbidity, and mortality [25,37-40]. The 3 French City Study, for example, found frailty to be significantly associated with a 4-year incidence of disability in ADL and IADL after adjustments [37].

While the strength of associations concur, the magnitude of the associations differ from study to study – inevitably due to study design, sample size, study populations’ health characteristics at baseline and specific conditions that define the sample population parameters such as inclusion and exclusion criteria, and particular to this study, the specific metrics used to operationalize the frailty definition. For example, in comparison to studies mentioned above, the SHARE population reported higher baseline burden of frailty, disability and morbidity; its eligibility criteria did not exclude cognitively impaired or terminally ill subjects; its sample was composed of both men and women, and the follow up time was 2 years. However, it also shared some similarities with the studies above, such as: a large sample size of community dwelling older adults aged 60 and older; a prospective follow up and use of the widely accepted frailty phenotype construct proposed by Fried and colleagues.

In spite of similarities and differences in study scope, design, and population characteristics, the heterogeneity in the prevalence of frailty remains largely a function of the operationalization and assessment of each criteria that defines the phenotype, which is mostly likely to differ from survey-to-survey due to the difference in its conceptualization, language formulation and measurement [18,37,40-46]. For example, the SHARE operationalization of “weakness” as measured by grip strength differed from that in the InChianti study conducted in Italy [40] and the Toledo Study on Health and Aging study conducted in Spain [47] in that both of these studies used their own lowest quintile cut-offs for their underlying population, adjusted for sex and BMI, compared to the sex and BMI specific cut points used in the original CHS population set by Fried [1]. In another instance, the absence of available data on grip strength prompted the use of a self-reported question on the difficulty in rising from a chair as proxy measure for the weakness criterion [37,38]. These discrepancies may explain some of the observed differences in frailty prevalence found in surveys conducted in Italy (8.8%) [40], Spain (8.4%) [47], and France (7%) [37]. Furthermore, in a German cohort, Saum and colleagues demonstrated a lower overall prevalence of frailty when using population or country specific lowest quintiles (6.5%) compared to population-independent cutoffs (8.9%) [48]. To reduce the heterogeneity in reporting frailty, standardization of cut points for the phenotype criteria may be the best viable option for making comparisons across culturally, economically, and socially diverse populations such as those found in SHARE countries more meaningful, especially since the association with adverse health outcomes has been validated in many countries.

This study found an association between frailty and worsening of morbidity, as measured by the deterioration in the number of select chronic conditions with respect to baseline. While a few researchers have used “worsening” as a metric to better understand the dynamic relationship between frailty and disability, it has been largely in relation to functional status [39]. SHARE data showed that while the odds ratio for worsening of morbidity was smaller than that for functional disability, it was not negligible. Moreover it showed significant values for prefrail individuals with slightly reduced strength of association. Although the odds ratios for the incidence and worsening models are of similar magnitude, the confidence intervals of the point estimates for worsening models are more narrow than that for incidence models, revealing more precise estimates – which may be due to the use of a larger analytical sample than one limited to a subset of those free of disability at baseline. Under resource and sample size constraints, a more dynamic metric such as “worsening” functional disability and worsening morbidity might serve to be more efficient.

This data further delineates the significant difference between frailty and prefrailty. In addition to the non-negligible difference in magnitude between frailty entities across all adverse outcomes, the observed data suggests presence of a nonlinear relationship - arguing for examining frailty on a continuous scale to better explore its dynamic and interdependent properties.

The major strength of this study is first, the reference to the widely adopted frailty phenotype, a construct that has a sound theoretical basis showing good content, construct, concurrent and predictive validity across diverse populations in both clinical and community settings [34,41]. Second, while the variables selected in SHARE depart from the original frailty criteria operationalized in the CHS study, this study attempted to mirror as many ‘original’ definitions in order to ensure valid comparisons as well as minimize any potential differential misclassification of outcome status. Three examples are highlighted: the use of the same chronic condition list, the incorporation of all 15 functional status items to assess disability, and the use of the same BMI and gender specific cut points to measure grip strength, all of which were established as per Fried et al. in the original CHS study. Third, the participation of 2 waves has lent itself in creating a longitudinal dimension allowing for a prospective analysis to be possible. Highly significant odds ratios have been found in spite of a relatively short 2-year follow up interval. Fourth, the risk of incident and worsening of functional status and morbidity is present even among the prefrail, after adjustments. Results suggest a possible gradient effect of frailty on adverse outcomes where changes in as little as 1 or 2 frailty criteria can impact on destabilizing a carefully balanced homeostatic physiological state into a vulnerable one. Fifth, the study’s large and diverse population-based sample covering 11 European countries of wide economic, social and cultural heterogeneity ensures greater generalizability to the community-dwelling Europeans aged 60+ as well as greater certainty around the point estimates. Lastly, this study includes a new metric: “worsening” of morbidity. “Worsening of morbidity”, as an alternative to “incidence of morbidity”, is a useful outcome to consider for both prevention and treatment strategies in aging population where chronic diseases dominate the health burden.

However a number of limitations must be made note. First, this study reports a substantial proportion of attrition. This is not an uncommon finding in large population-based, multi-country, prospective studies. The analyses of baseline characteristics of the untapped data (lost to follow-up, refusals, deaths) reveal that this group tended to be younger (65 vs. 70), while sex status was non-differential. This group was only slightly more prefrail or frail (49%) compared to those in the analytical sample (45%). Attrition may potentially lead to a selection bias that might have spuriously resulted in a “healthy aging” effect. However, due to small differences, it is unlikely to compromise the interpretation of the results. Second, while the definition of morbidity used in this study was directly adopted from the CHS to show alignment and comparability with Fried et al., the measure is somewhat limited in the number and type of chronic diseases that it contains. For example, it omits prevalent conditions encountered in older persons such as dementia, degenerative arthritis, and mental health conditions. In contrast, this study used broad and rather non-specific criteria defining mobility disability consistent with other studies examining the relationship between frailty and disability [1,37,38]. The incidence of mobility disability among the frail, prefrail and non-frail groups in SHARE demonstrated to be of similar magnitude across the frailty gradient of that found in 3 City French study: frail (70.5% vs 68%), prefrail (43% vs 55%) and non frail (29% vs 45%) [37]. And finally, inherent to all survey data is the reliance on “self-reported” information which is limited by its inability for independent verification. Self-reported data pose differential misclassification bias by sex, country or health status, and must be taken into account when interpreting results. In general, caution must be taken when making comparisons across countries, in that the variability in time, place and people will always exist.

## Conclusions

The SHARE-operationalized frailty phenotype has established good construct validity by showing significant association with adverse outcomes, independent of baseline disability and comorbidity in the European population aged 60 and older. These findings provide fertile ground for investigating more complex interactions of biological, environmental and social circumstances that impact on heath trajectories, and shape the heterogeneity of frailty in aging populations. SHARE, with its wide-scope and detail prospective data on individual, economic, social, and environmental and health characteristics, is the ideal medium to further epidemiological research on frailty and health in Europe.

## Abbreviations

CAPI: Computer assisted personal interview technique; CHS: Cardiovascular Health Study; SHARE: Survey of Health Aging and Retirement in Europe; BADL: Basic activities of daily living; IADL: Instrumental activities of daily living; ADL: Activities of daily living; HBP: High blood pressure; PPP: Purchasing power of parity; OR: Odds ratio; WHIOS: Women’s Health Initiative Observation Study; WHAS I & II: Women’s Health and Aging Study I and II.

## Competing interest

Authors declare that they have no competing interests.

## Authors’ contributions

NM conceived the study objectives, performed the data analysis, interpreted the data and drafted the manuscript. JJ prepared the database, and with JS provided statistical support on STATA. BSE supervised the conceptualization of the study and analytical strategy, and participated in the revisions of the manuscript. All authors read and approved the final manuscript.

## Pre-publication history

The pre-publication history for this paper can be accessed here:

http://www.biomedcentral.com/1471-2318/13/3/prepub
